# Supplementary material for: Differential responses of primary neuron-secreted MCP-1 and IL-9 to type 2 diabetes and Alzheimer’s disease-associated metabolites
Source: Sci Rep. 2024 Jun 3;14:12743. doi: 10.1038/s41598-024-62155-3 (PMC11148169; doi:10.1038/s41598-024-62155-3)
Supplement: Supplementary file 1 — Supplementary Information 1. [file 41598_2024_62155_MOESM1_ESM.pdf]

Supplemental Information

Differential responses of primary neuron-secreted MCP-1 and IL-9 to type 2 diabetes and Alzheimer's disease-associated metabolites

Authors and Affiliations: Brendan K. Ball[1], Madison K. Kuhn[2-5], Rebecca M. Fleeman Bechtel[2-3], Elizabeth A. Proctor[2-6], Douglas K. Brubaker[7-8*]

1. Weldon School of Biomedical Engineering, Purdue University, West Lafayette, IN, USA
2. Department of Neurosurgery, Penn State College of Medicine, Hershey, PA, USA
3. Department of Pharmacology, Penn State College of Medicine, Hershey, PA, USA
4. Department of Biomedical Engineering, Penn State University, State College, PA, USA
5. Center for Neural Engineering, Penn State University, State College, PA, USA
6. Department of Engineering Science & Mechanics, Penn State University, State College, PA, USA
7. Center for Global Health & Diseases, Department of Pathology, School of Medicine, Case Western Reserve University School of Medicine, Cleveland, OH, USA
8. Blood Heart Lung Immunology Research Center, University Hospitals, Cleveland, OH, USA

***Corresponding Author:** dkb50@case.edu

SUPPLEMENTARY FIGURES

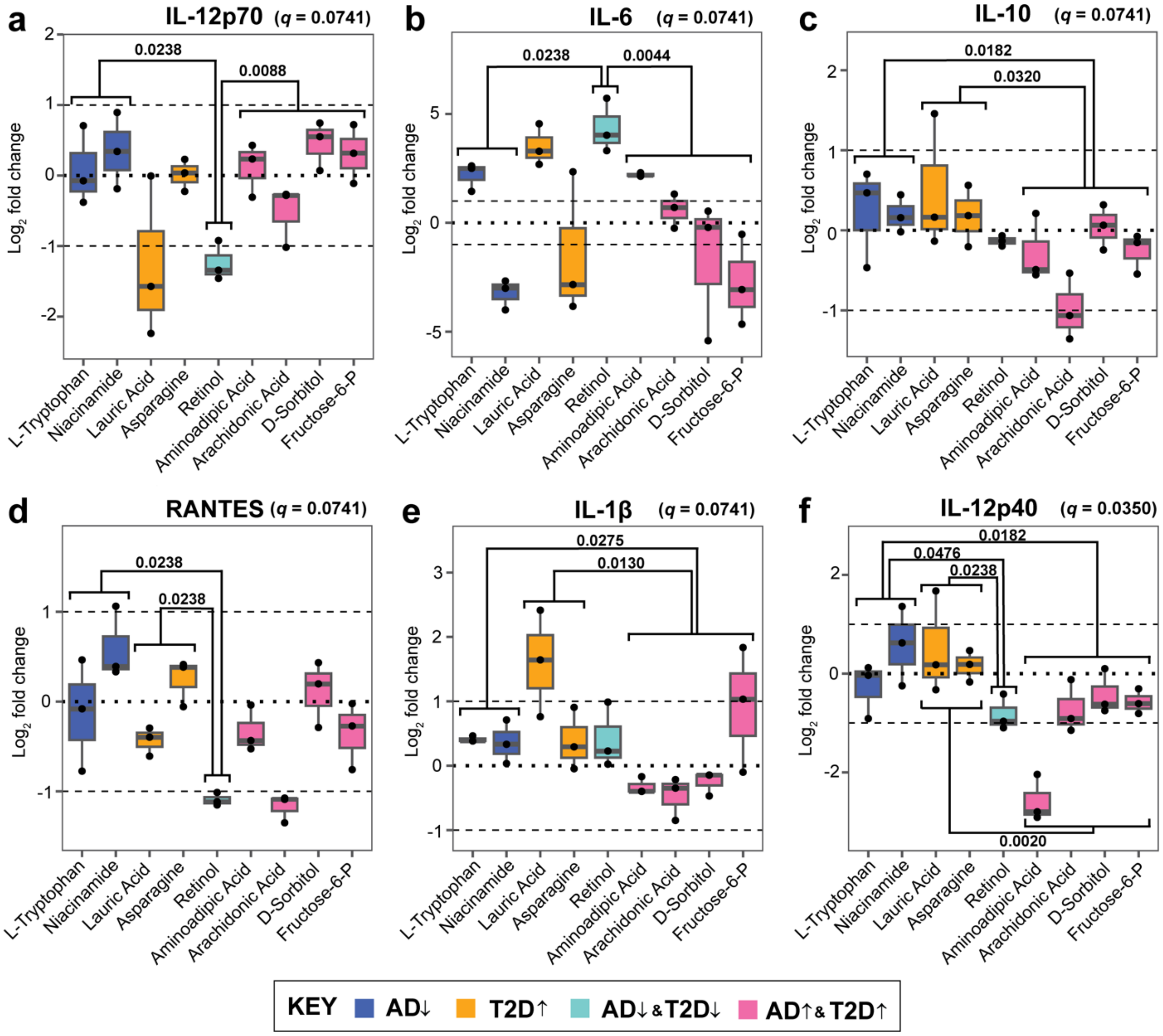

Supplementary Figure S1. Additional Significantly Reported Results on Neurons treated with different disease associations. The $\log_2$ ratio of cytokine concentration to vehicles that were determined significant from a Kruskal-Wallis test (FDR *q* value next to the cytokine). Mann-Whitney pair-wise testing was applied to each metabolite group based on disease association (significance denoted within the plot). The cytokines include **(a)** IL-12p70, **(b)** IL-6, **(c)** IL-10, **(d)** RANTES, **(e)** IL-1β, and **(f)** IL-12p40.

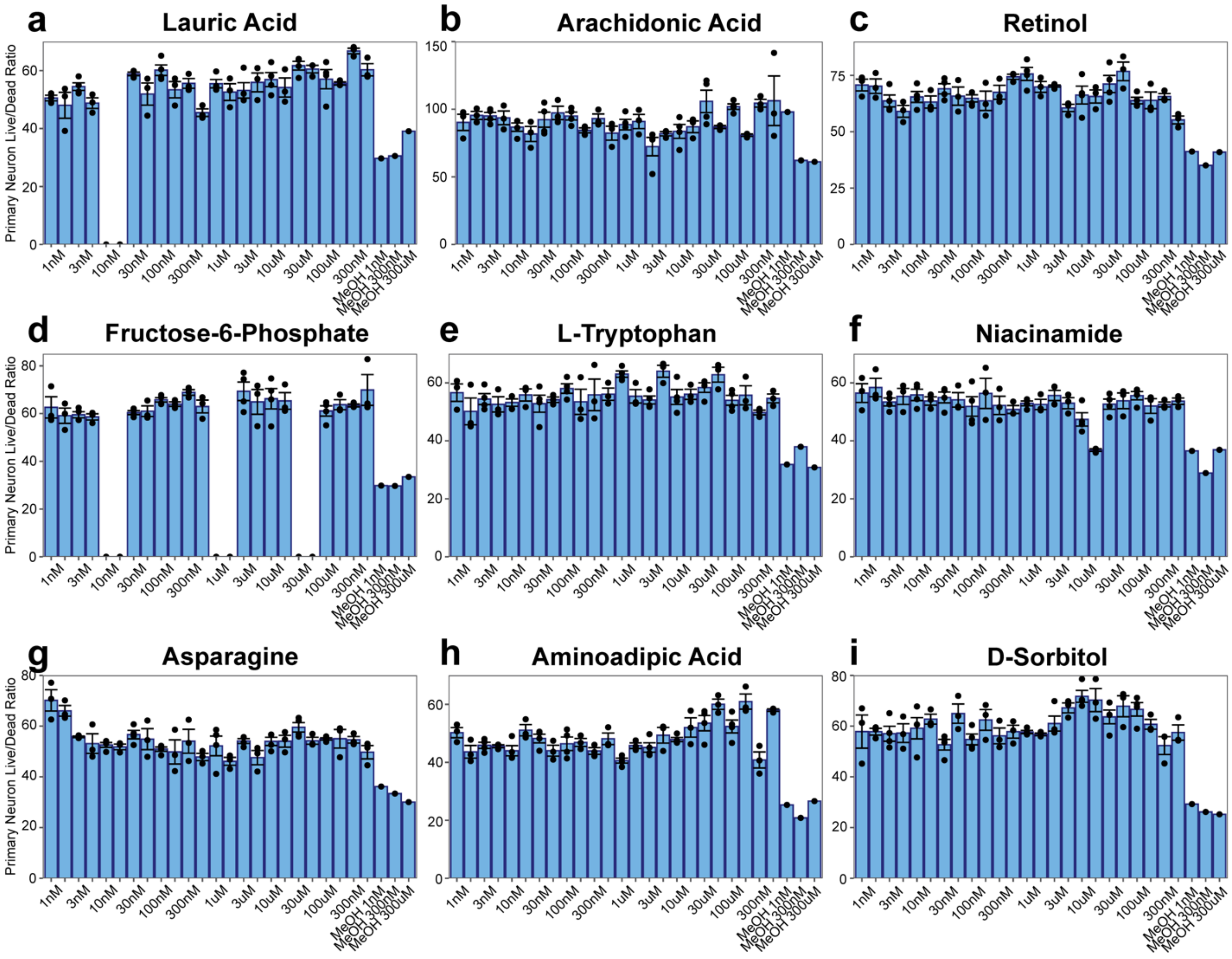

Supplementary Figure S2. Live-dead ratio of each metabolite stimulation on primary neuron culture. Live-dead assay was performed on **(a)** lauric acid, **(b)** arachidonic acid, **(c)** retinol, **(d)** fructose-6-phosphate, **(e)** L-tryptophan, **(f)** niacinamide, **(g)** asparagine, **(h)** aminoadipic acid, and **(i)** D-sorbitol. Per each concentration, the metabolite (left-side bar) and vehicle (right-side bar) is reported. Missing data is due to extreme outlier (lauric acid) and limitation of primary neurons (fructose-6-phosphate). Live-dead ratio is displayed as mean ± standard error of the mean.

SUPPLEMENTARY TABLES

Supplementary Table S1. Evidence of studied systems for each metabolite

Metabolite	Association	System	Relation to Disease	Ref.
Lauric Acid	T2D$^{\uparrow}$	Mouse Liver	**Study:** 7-week old C57BL/6J male mice were fed with standard chow, with lauric acid dissolved in drinking supply. Mice were orally administered lauric acid or same dose vehicle once every day for 6-weeks, or twice a day for 5-weeks. **Results:** Lauric acid resulted in downstream hepatic insulin resistance.	[43]
Lauric Acid	T2D$^{\uparrow}$	Human Plasma	**Study:** A longitudinal human study of 106 participants for 4 years collecting multi-omics data of healthy and people with pre-diabetes. **Results:** Lauric acid was found as a metabolite upregulated in people with diabetes/pre-diabetes than healthy groups upon inspection of publicly available data sets.	[42]
Asparagine	T2D$^{\uparrow}$	Human Plasma	**Study:** Clinical study comparing 1032 human participants with T2D and 1522 participants without T2D. Serum asparagine and aspartate levels were quantified. **Results:** A ratio of asparagine to aspartate greater than 1.5 was correlated with elevated T2D risk.	[44]
Asparagine	T2D$^{\uparrow}$	Mouse Brain	**Study:** Male control and AD (APP/PS1) mouse groups were aged at 6, 12, and 24 months of age and were tested in a 3-shock contextual fear conditioning for memory assessment. After the study, the prefrontal cortex, hippocampus, and spleen were collected for metabolomics. **Results:** Asparagine was positively correlated to freezing behavior in the prefrontal cortex of AD (APP/PS1 mouse) groups.	[45]

Fructose-6-Phosphate	AD$^{\uparrow}$, T2D$^{\uparrow}$	Rat Blood, Liver	**Study:** Adult male Sprague-Dawley rats were either fed a control or high-fructose diet for 19 weeks. Using a spatial water maze, cognitive function was quantified by the latency to reach a platform. Blood plasma and the liver were collected for analysis. **Results:** Rats fed with a high fructose diet demonstrated significantly longer latencies to reach the platform than control groups, suggesting that there may be some maze retention performance between the two groups.	[46]
Fructose-6-Phosphate	AD$^{\uparrow}$, T2D$^{\uparrow}$	Rat Blood, Pancreas and Liver	**Study:** Male Wistar rats (10-12 weeks old) were divided into four different groups – normal diet, high-fructose diet, high-fat diet, and high-fructose + high-fat diet. All diets were fed for 4 months. Blood samples and organs were collected for biochemical markers and mRNA/protein analysis, respectively. **Results:** Experimental support that diets composed of high-fructose also result in detrimental metabolic disorder.	[47]
Arachidonic Acid	AD$^{\uparrow}$, T2D$^{\uparrow}$	Mouse, Brain, Cell Line	**Study:** Male mice were placed on 1 of 3 dietary groups (n=5 each) with arachidonic acid. At 23-24 weeks of age, the mice were sacrificed and sectioned sagittal in hemibrains for immunohistochemistry and image analysis. For cell studies, COS-7 cells transfected with pCEP-SP-C99 (encoded with human C-terminal fragment APP; APP-C99 plasmids) were stimulated with various ranges of arachidonic acid to measure amyloid-beta activities. **Results:** Cell culture experiments suggested that arachidonic acid and other metabolites driven the altered production of amyloid-beta through gamma-secretase	[48]

			cleavage of amyloid precursor protein. Mouse studies supported the *in vitro* findings by showing elevated amyloid-beta levels in the brain in groups with arachidonic acid supplemented chow.	
Arachidonic Acid	AD$^{\uparrow}$, T2D$^{\uparrow}$	Human Adipose Tissue, Blood Sample	**Study:** 484 participants were examined for the association between adipose tissue biopsy, fasting blood sample, arachidonic acid, and metabolic syndrome in Costa Rica (criteria based on the National Cholesterol Education Program Expert Panel). **Results:** Multivariate logistical analysis determined that greater adipose tissue arachidonic acid levels had an increased risk for metabolic syndrome (conditions consisted of heart disease, stroke, and diabetes). Arachidonic acid is an important independent marker of metabolic dysregulation.	[49]
Aminoadipic Acid	AD$^{\uparrow}$, T2D$^{\uparrow}$	Mouse Brain	**Study:** Male control and AD (APP/PS1) mouse groups were aged at 6, 12, and 24 months of age and were tested in a 3-shock contextual fear conditioning for memory assessment. After the study, the prefrontal cortex, hippocampus, and spleen were collected for metabolomics. **Results:** Aminoadipic acid was positively correlated to freezing behavior in the hippocampus of AD (APP/PS1 mouse) groups.	[45]
Aminoadipic Acid	AD$^{\uparrow}$, T2D$^{\uparrow}$	Human Blood, Mouse Blood, Cell Lines	**Study:** Plasma samples collected from two separate human cohorts for a discovery analysis and replication analysis from a FHS Offspring Study and Swedish population-based cohort, respectively. Fasting plasma samples were also collected. Polar plasma metabolites were collected using hydrophilic interaction liquid	[50]

			chromatography. Mouse groups with different diets as well as human/mouse islets (for insulin secretion) studies were also included in the study. **Results:** Aminoadipic acid was identified as a biomarker predictive of diabetes in healthy individuals. Two independent human cohorts show that high plasma aminoadipic acid levels resulted in a 4-fold risk for the development of T2D.	
D-Sorbitol	AD$^{\uparrow}$, T2D$^{\uparrow}$	Mouse Brain	**Study:** Male control and AD (APP/PS1) mouse groups were aged at 6, 12, and 24 months of age and were tested in a 3-shock contextual fear conditioning for memory assessment. After the study, the prefrontal cortex, hippocampus, and spleen were collected for metabolomics. **Results:** Sorbitol was positively correlated to freezing behavior in the hippocampus of AD (APP/PS1 mouse) groups.	[45]
D-Sorbitol	AD$^{\uparrow}$, T2D$^{\uparrow}$	Mouse Blood	**Study:** Comparison of the differential effects of short-term and long-term consumption of sorbitol in mice. C57BL/6 mice at 6-weeks old were administered with sorbitol or vehicle by oral gavage for 4 weeks. After the 4 weeks, oral glucose tolerance tests, intraperitoneal glucose tests, and insulin level tests were performed. Fecal pellets were collected for 16s rRNA sequencing to determine gut microbiome changes. **Results:** Long-term sorbitol administration via oral gavage induced glucose intolerance and alteration of the gut microbiome in mice.	[51]3/6/24 3:53:00 AM
Retinol (Vitamin A)	AD$^{\downarrow}$, T2D$^{\downarrow}$	Human Plasma	**Study:** A longitudinal human study of 106 participants for 4 years collecting multi-omics data of healthy and people with pre-diabetes. **Results:** Retinol was	[42]

			found as a metabolite downregulated in people with diabetes/pre-diabetes than healthy groups upon inspection of publicly available data sets.	
Retinol (Vitamin A)	AD↓, T2D↓	Human Brain	**Study:** Frozen samples of human brain were dissected into white and grey matter. A total of 10 healthy and 21 AD post-mortem brains were used in the study and analyzed by high-performance liquid chromatography. The study compared micronutrient levels between both cases. **Results:** Retinol was found to be in significantly decreased levels in AD brains than healthy brains.	[52]
L-Tryptophan	AD↓	Human Plasma	**Study:** Assessed cognitive function and mood of a reduction of brain serotonin by acute tryptophan depletion. The study included 16 participants with AD and 16 participants without AD. Participants received a tryptophan-free amino acid drink or placebo drink with balanced amino acids. Ratings of mood and Modified Mini-Mental State scores were recorded. **Results:** Participants with AD had a significantly lower Modified Mini-Mental State than healthy participants after acute tryptophan depletion.	[53]3/6/24 3:53:00 AM
L-Tryptophan	AD↓	Mouse Brain	**Study:** 2-month-old male 3xTg and non-3xTg mice were each assigned to three different dietary groups (low, normal, and high tryptophan). After 1 month of the diet, the mice were perfused and prepared for quantification of CA1 serotonin transporter fiber density, amyloid beta deposition, as well as the total count of serotonergic neurons. **Results:** Increased dietary intake of tryptophan reduced intraneuronal amyloid beta in the hippocampus,	[54]

			suggesting that tryptophan may reduce AD pathology.	
Niacinamide	AD↓	Mouse Brain, Human Plasma	**Study:** Hippocampi of 9-month-old male and control rat models (transgenic McGill-R-Thy1-APP) were collected and homogenized for untargeted nuclear magnetic resonance microscopy. Metabolomic profiles were measured to compare between groups. Human plasma samples were selected from the Germany study on Aging, Cognition, and Dementia biobank (longitudinal study). Neurological assessment to determine conversion to AD condition and nicotinamide levels in blood were reported. **Results:** Niacinamide was one of the metabolites selected from a PLS-DA model with a higher VIP score of 9 significant metabolites. High plasma levels of nicotinamide in humans showed a 27% risk reduction in AD development.	[56]
Niacinamide	AD↓	Mouse Brain	**Study:** Triple transgenic (3xTg) AD and control mouse groups (n=8/group) at 4-months of age were orally given niacinamide with behavioral studies (memory and fear conditioning) at 8 and 12 months of age. Confocal microscopy for amyloid beta and tau was also conducted. **Results:** Oral treatment of niacinamide was found to prevent cognitive deficits in mild to moderate AD mouse models.	[55]

Supplementary Table S2. Verification of Blood-Brain Barrier Permeability

Metabolite	Association	Brief Overview of Published Study	Ref.
Lauric Acid	T2D$^{\uparrow}$	In silico study on different compounds and passage of the BBB in the context of Tuberculosis Treatment.	94
Asparagine	T2D$^{\uparrow}$	Cerebrospinal fluid asparagine levels of children with acute lymphoblastic leukemia.	95
Fructose-6-Phosphate	AD$^{\uparrow}$, T2D$^{\uparrow}$	Regional cerebral blood flow of fructose to cross the BBB into the hypothalamus.	96
Arachidonic Acid	AD$^{\uparrow}$, T2D$^{\uparrow}$	Association and influence on increased BBB permeability.	97
Aminoadipic Acid	AD$^{\uparrow}$, T2D$^{\uparrow}$	Precursor in the biosynthesis of lysine, with discussion on amino acids crossing the BBB.	101
D-Sorbitol	AD$^{\uparrow}$, T2D$^{\uparrow}$	Study on an intermediate molecule (sorbitol) in the polyol pathway. Opens the possibilities of D-Sorbitol involvement in sugar transport.	114
Retinol (Vitamin A)	AD$^{\downarrow}$, T2D$^{\downarrow}$	Transport across the BBB. Studies discuss the role of STRA6 to facilitate transport.	98
L-Tryptophan	AD$^{\downarrow}$	Review on tryptophan, properties, and passage through the BBB.	99
Niacinamide	AD$^{\downarrow}$	Review on niacinamide and ability to pass through the BBB.	100

Supplementary Table S3. Metabolite Concentration Ranges on Previously Published Literature

Metabolite	Association	Concentration	Cell Line Used	Ref.
Lauric Acid	T2D$^{\uparrow}$	50-100 uM	KT-5 astrocyte cell line	[103]
Asparagine	T2D$^{\uparrow}$	0.1-3 mM	HeLa (ATCC), A431, LPS2, and SUM159PT cells	[104]
Fructose-6-Phosphate	AD$^{\uparrow}$, T2D$^{\uparrow}$	277 mM	Neuroblastoma x mouse spinal cord motor neuron cell line (NSC-34)	[105]
Arachidonic Acid	AD$^{\uparrow}$, T2D$^{\uparrow}$	0.005-0.05 mM	Hep G2 -MV2E1-9 cell lines	[106]
Aminoadipic Acid	AD$^{\uparrow}$, T2D$^{\uparrow}$	0.21-0.34 mM	Monolayer culture of HimOF1(Swiss)SF mice cerebellum	[107]
D-Sorbitol	AD$^{\uparrow}$, T2D$^{\uparrow}$	0.5-1.5 M	Human HCT116 cell line (colorectal cancer cell)	[108]
Retinol (Vitamin A)	AD$^{\downarrow}$, T2D$^{\downarrow}$	10 uM	Adult neurons from *Lymnaea stagnalis*	[109]
L-Tryptophan	AD$^{\downarrow}$	25-50 uM	EG7-OVA (mouse T lymphoma line transfected with chicken OVA) and HeLa (epithelial carcinoma cell line)	[110]
Niacinamide	AD$^{\downarrow}$	10 mM	Mouse embryonic stem cells	[111]
